# What is needed for implementing drug checking services in the context of the overdose crisis? A qualitative study to explore perspectives of potential service users

**DOI:** 10.1186/s12954-020-00373-4

**Published:** 2020-05-12

**Authors:** Bruce Wallace, Thea van Roode, Flora Pagan, Paige Phillips, Hailly Wagner, Shane Calder, Jarred Aasen, Bernie Pauly, Dennis Hore

**Affiliations:** 1 Canadian Institute for Substance Use Research, University of Victoria, PO Box 1700, STN CSC, Victoria, BC Canada; 2 School of Social Work, University of Victoria, PO Box 1700, STN CSC, Victoria, BC Canada; 3 SOLID Outreach Society, 1056 North Park St, Victoria, BC Canada; 4 AVI Health & Community Services, 3rd Floor - Access Health Centre 713 Johnson St, Victoria, BC Canada; 5 Lantern Services, 820 Cormorant St, Victoria, BC Canada; 6 School of Nursing, University of Victoria, PO Box 1700, STN CSC, Victoria, BC Canada; 7 Department of Chemistry, University of Victoria, PO Box 1700, STN CSC, Victoria, BC Canada; 8 Department of Computer Science, University of Victoria, PO Box 1700, STN CSC, Victoria, BC Canada

**Keywords:** Drug checking, Overdose, Harm reduction, Spectroscopy, Substance use, Fentanyl, Implementation science, Consolidated Framework for Implementation Research

## Abstract

**Background:**

The current illicit drug overdose crisis within North America and other countries requires expanded and new responses to address unpredictable and potentially lethal substances, including fentanyl analogues, in the unregulated drug market. Community-wide drug checking is being increasingly explored as one such public health response. We explored how drug checking could be implemented as a potential harm reduction response to the overdose crisis, from the perspective of potential service users.

**Methods:**

The research was guided by the Consolidated Framework for Implementation Research (CFIR). We conducted a qualitative, pre-implementation study to inform development and implementation of drug checking services that are acceptable to people who use substances and meet their needs. University and community researchers conducted 27 in-depth interviews with potential service users at prospective drug checking sites. We inductively developed emerging themes to inform the implementation of drug checking services within the five domains of the CFIR, and identified the most relevant constructs.

**Results:**

Implementing community drug checking faces significant challenges within the current context of criminalization and stigmatization of substance use and people who use/sell drugs, and trauma experienced by potential service users. Participants identified significant risks in accessing drug checking, and that confidential and anonymous services are critical to address these. Engaging people with lived experience in the service can help establish trust. The relative advantage of drug checking needs to outweigh risks through provision of accurate results conveyed in a respectful, non-judgemental way. Drug checking should provide knowledge relevant to using and/or selling drugs and informing one’s own harm reduction.

**Conclusions:**

For service users, the extent to which the implementation of drug checking can respond to and mitigate the risks of being criminalized and stigmatized is critical to the acceptability and success of community drug checking. The culture and compatibility of the service, setting and staff with harm reduction principles and practices is essential.

## Background

The illicit drug overdose crisis continues in Canada. One person lost their life to overdose approximately every two hours in Canada in 2018 [1]. The province of British Columbia (BC) is considered the epicentre of the crisis where the rate of illicit drug overdose deaths has reached 31 per 100,000 individuals, an average of four people dying each day [2]. Illicit fentanyl has been detected in 87% of these drug overdose deaths in the province [3]. In 2016, the escalating rate of overdose deaths in BC triggered the Provincial Health Officer to declare illicit drug overdoses a public health emergency [4, 5] which is still in effect.

It is within this context of fentanyl-related overdoses that drug checking has been re-emerging as a potential harm reduction intervention in Canada [6–8] and globally [9–11]. A 2017 evidence review of drug checking technologies and research concluded that community-wide drug checking may be instrumental as part of public health responses in British Columbia, and potentially a life-saving harm reduction service [12]. At the same time, caution is advised as “[I]mplementation in the absence of rigorous evaluation could result in the wasting of precious resources, and more importantly, more lost lives to fatal overdose” [13] (p. 2).

The body of research related to drug checking is primarily based on the long-standing history of drug checking within electronic dance and festival settings. The relevance of this research for community-wide drug checking that serves the general population and responds to illicit drug toxicity, including fentanyl-related overdose, is in question [11, 12, 14, 15]. The willingness among people who inject drugs to access community drug checking has also been questioned [16]. Limited uptake of this intervention has been reported in some cases [17], and potential strong acceptance in others [18–20]. Early research on community drug checking for street drugs raises questions about whether or how drug checking results will modify behaviours [16], as well as which drug checking technologies are best in these settings and for which substances and populations [12, 14, 16, 21].

While many harm reduction interventions have a well-established evidence base as to their effectiveness [22–28], as Rhodes and others [29] have argued, how harm reduction interventions are implemented and the context for implementation of such services matters greatly. Moreover, consistent with principles of harm reduction, it is critical that people who use substances are engaged in the design and delivery of services intended for them, in order to ensure that services are acceptable and meet their needs. The structural contexts in which substance use occurs, such as criminalization and structural violence, and the way harm reduction interventions are enacted demand attention if harm reduction services are to be effective in reducing harms beyond individual level, behaviour modifications [30, 31]. Thus the evidence base regarding community-wide drug checking as a response to the overdose crisis is still emerging [11], and there is limited focus on implementation research that involves people who use substances beyond the dance and festival settings. Such research is needed to support implementation of drug checking services as a harm reduction response that takes the context of the overdose crisis into account, from the critical perspective of people most affected by it.

We sought to explore how drug checking could be implemented as a potential harm reduction response to the overdose crisis, from the perspective of potential service users. This research was conducted as part of the process of developing and initiating a community drug checking project within Victoria, BC, where the burden from the overdose crisis has been extremely high and drug checking services were not previously available. We conducted a pre-implementation study to inform the development and implementation of drug checking services that are acceptable to people who use substances and meet their needs.

## Methods

This study was part of a community drug checking project “Implementing Innovations in Drug Checking” that aims to offer drug checking services within Victoria, BC, improve methods and technologies for drug checking and understand factors that influence implementation. As part of the broader study, we conducted a pre-implementation qualitative study. Our collaborative research team included academic researchers and community researchers from the local drug user union Solid Outreach. Ethical approval for the study was provided by the Health Research Ethics Board at Island Health Authority (J2018-069).

We conducted this research within Canada’s Strategy for Patient-Oriented Research (SPOR) [32] which supports meaningful involvement of patients, researchers, health care providers and decision-makers in health research. While levels of engagement vary within Patient-Oriented Research (POR) [33], we sought collaborative partnerships and to enact principles of Community-Based Participatory Research (CBPR) by seeking to engage individuals with personal experience of a health issue in the research process. CBPR is an approach that engages community equitably in the research process for the purpose of producing knowledge and taking action [34, 35]. The academic researchers have had long-standing relationships with the community-members we engaged in this project, and these relationships are ongoing beyond the data collection and the production of this article [36]. Indeed, we perceive the onus for participation to be focused on academic researchers participating in community just as much as we seek engagement of community members in this research [37].

We used the Consolidated Framework for Implementation Research (CFIR) [38] to guide identification of potential barriers and facilitators for implementation and explore the unique context for implementation of drug checking services. CFIR has been shown to be relevant to understanding what is needed for successful implementation overall and to the implementation of public health interventions. The CFIR framework consists of 39 theoretical concepts or constructs grouped into five domains capturing determinants affecting implementation of an intervention at the organizational or collective level [38]. The five domains include (1) characteristics of individuals receiving and delivering the intervention, (2) characteristics of the intervention (in this case community drug checking), (3) the process of implementing community drug checking, (4) the inner setting (e.g. policies, organizational culture, funding) and (5) outer setting (funding, policies, laws, etc.). Here we used the CFIR as part of the pre-implementation phase of the drug checking project. In a systematic review of 26 studies using the CFIR framework, Kirk et al. [39] found only 2 studies that focused on pre-implementation, naming it a missed opportunity. While the CFIR has been used most often during or post-implementation as a pre-implementation framework, it provides unique opportunities to identify potential barriers in advance and inform project design so that services are acceptable to service users and meet their needs. In this project, the research team is considered to be the implementer of the intervention and our pre-implementation study sought the perspectives of potential service users to inform implementation plans.

Within a framework of community-based research, it is possible to employ a range of research methods. In this study, we undertook a qualitative study with potential service users. The inquiry was a collaboration between community researchers from the local drug user union and university-based researchers. The university-based researchers developed a semi-structured qualitative interview guide to seek perspectives on potential barriers and facilitators across each of the five domains of the CFIR. The community researchers revised this questionnaire to better suit research participants and to ensure we approached questions about substance use with respect and recognition of the lived realities of stigma and criminalization. We also developed a short survey to collect information on demographics and substance use which community researchers reviewed. Pilot interviews were conducted with members of the drug user organization to refine the data collection process.

Interviews were open to people who use or have used substances, their family or friends and/or people who make or distribute substances. Recruitment of research participants occurred at the harm reduction sites proposed as locations for implementing and integrating the upcoming drug checking services. This included a drug user organization that provides services by and for people who use drugs, a community harm reduction site that includes a drop-in medical clinic and needle exchange programme as well as other services, and a suburban harm reduction site offering opioid substitution therapy along with other services. Handbills and posters were distributed at the sites to potential participants, and an email invite was distributed to the staff at the sites to inform them of the study and information to share with potential participants. Study participants were provided CDN$20. Interviews were conducted by either a community or university researcher and audio-recorded and transcribed verbatim.

### Analysis

We collected data from November 2018 to February 2019. The principal investigator and two research associates reviewed, coded and compared initial themes that inductively emerged on a small subset of transcripts. Based on this and feedback from interviewers on any themes that they detected from the interviews, we then developed an initial coding structure to analyse potential barriers and facilitators for drug checking services, from the perspective of people who use substances and other potential service users, within each of the five domains of the CFIR. Two research associates coded all transcripts into this initial structure in NVIVO 11. Initial coding of a subset of transcripts was coded by both research associates, reviewed with the Principal Investigator and revised accordingly. The research associates then coded the remaining transcripts. The Principal Investigator and research associates then inductively developed critical themes to inform the development and implementation of drug checking services for each of the five domains of the CFIR and identified the most relevant constructs associated with these themes. All authors including those involved in the interview process reviewed the analysis and interpretation. For each domain, we present the themes that emerged within that domain. As we found significant contextual barriers in the outer setting that need to be attended to in all other domains, we present the outer setting first.

## Results

There were 27 people who participated in interviews at three different sites: 14 interviews at the drug user organization that provides services by and for people who use drugs, 7 interviews at a community harm reduction site that includes a drop-in medical clinic and needle exchange programme as well as other services, and 6 interviews at a suburban harm reduction site offering opioid substitution therapy along with other services. About half (*n* = 14) of the participants identified as men and most (*n* = 14) participants were over the age of thirty. The sample was predominantly individuals with low-incomes with most (*n* = 20) receiving social assistance and over half (*n* = 15) living in supportive or subsidized housing, single room occupancy or rooming houses or currently homeless. Characteristics of the sample are given in Table 1.

**Table 1:** Characteristics of the sample (*N* = 27)

Characteristic	Number (percent)
	***n*** (%)
**Gender**^**a**^	
Non-binary/transgender/other^d^	0 (0.0)
Man	14 (51.9)
Woman	13 (48.2)
**Age in years**^**abcd**^	
20–29	4 (22.2)
30–44	6 (33.3)
45 or older	8 (44.4)
**Identified as Indigenous (First Nations, Métis or Inuk (Inuit))**	
No	23 (85.2)
Yes	4 (14.8)
**Sexual orientation**^**a, b, c, d**^	
Heterosexual or straight	14 (82.4)
Lesbian or gay/two-spirit/queer/bisexual/other/do not know	3 (17.6)
**Current level of education**^**a, b, c, d**^	
Some secondary education	3 (16.7)
High school diploma or equivalency	5 (27.8)
Apprenticeship, trades certificate or diploma, other certificate, diploma or degree	6 (33.3)
Other	4 (22.2)
**Current living situation**^**a, c, d**^	
Apartment/house	9 (33.3)
Supportive or subsidized housing/single room occupancy or rooming house	9 (33.3)
Public place or street	6 (22.2)
Family or friend’s place, other	3 (11.1)
**Primary source of income**^**d**^	
Wage or salary including from own business	4 (14.8)
Disability benefits	16 (59.3)
Welfare or income assistance	4 (14.8)
Other	3 (11.1)
**Personal income last year**^**a, c, d**^	
Less than $20,000	13 (50.0)
$20,000 to less than $40,000	7 (26.9)
$40,000 or more	3 (11.5)
Do not know	3 (11.5)
**Frequency of illicit substance use**^**d**^	
Daily	19 (70.4)
Three or more times per week	2 (7.4)
Once or twice per week	3 (11.1)
Occasionally (not every month) or Never	3 (11.1)
**Overdose within last 6 months**^**c**^	
No	20 (76.9)
Yes	6 (23.1)

^a^Does not sum to 100.0% due to rounding

^b^Demographic questions were altered. Not asked of 9 study members in early interviews

^c^Does not sum to 27 due to missing data

^d^Some categories were combined due to small numbers to preserve anonymity

### Outer setting

Participants shared their perspectives on what contextual factors could have positive or negative influences on the implementation of drug checking services. This revealed significant influences in the outer setting that need to be considered in every aspect of implementation. The need for drug checking was related to the unregulated, unpredictable and potentially risky illicit drug market. Implementation of drug checking must recognize the needs of the diverse population that uses substances and account for the very significant impacts of stigma, trauma and criminalization on service users.

#### Diverse population and needs

Participants emphasized that substance use occurs across all ages, genders, socioeconomic groups and geographic areas. They noted that potential service users have varied needs and resources and services should be tailored to best respond to these diverse needs. For example, one participant indicated that some may not be able to access services easily: “There’s lots of people using who just can’t get to the stuff that we can get to downtown. It’s just not available to them. They don’t drive or don’t have bus fare. They’re using and they need that (INTV F2)”*.* Further, this quote highlights the diverse population that uses substances and that services may need to be different for different groups:

Everybody uses drugs. Or not everybody, but you know, there’s business people out there, there’s lawyers out there that use drugs They’re not going to go to a place that let’s say people who are homeless would go to, unfortunately, due to the stigma. Which sucks, because there shouldn’t be a stigma, but there is (INTV F17).

The above quote also illustrates that it is important not to make assumptions about people who use substances.

For some potential service users, substance use may be associated with a number of difficult experiences such as homelessness, poverty, overdose, death of a friend or family member to overdose, chronic pain or traumatic brain injury. For example, this participant notes that the experience of overdose has impaired their ability to function as they used to: “I’ve had so many ODs in my life that my brain doesn’t work as good as it used to, and I’ve forgotten lots of stuff that I’ve learned so I don’t understand a lot of things (INTV F1)”*.* Furthermore, people’s attitudes and experiences around substance use vary substantially. This includes how much experience they have had with substances, whether they use alone or with others, whether they are looking to hide their use, seek treatment for use, are using harm reduction services, and/or practicing their own harm reduction measures to stay safe. This participant discussed that those who use alone do so because they do not want others to know about their use, or because they do not have a way to access services:

I know that there are some people like that, who are still in the closet and don’t want anyone to know, they’re embarrassed or ashamed or whatever, but the people I know that are using at home, they’re only using alone at home because they have no way to get to services, or they would, they would have it checked for sure. I know both groups and the other groups that are at home, they just don’t want their families to know, so they’re not going to come in and use the services and get them checked. They just don’t want anyone to know (INTV F2).

Overall, these findings highlight that it is critical not to make assumptions about who uses substances. It is essential to recognize that substance use is common across all demographic groups, constituting an extremely diverse population with diverse needs that needs to be taken into account. Thus, participants identified significant structural factors that must be recognized, and implementation strategies are needed that attend to these critical barriers.

#### Criminalization

The criminalization of drugs and people who use and/or sell drugs was the most significant influence from the perspective of potential service users. Participants described how they and others often feared police presence and prosecution or any level of surveillance, as this participant expressed: “Well, if the cops are going to be hanging around, if there’s a chance of getting legally busted or charged, obviously that would be first and foremost (INTV F3)”. There were related concerns that drug checking services might be used by police to gain information on who is selling and using and what substances they had or to look for particular individuals. For example, one participant stated this would be a barrier: “Well, if the cops are going to be hanging around, if there’s a chance of getting legally busted or charged, obviously that would be first and foremost (INTV F3)”. A potential facilitator identified by participants would be government sanctioning of drug checking to establish safety from criminal repercussions, with recommendations that decriminalization of drugs would be the ultimate facilitator to influence implementation. This quote begins to illustrate the impact of criminalization and the need for government involvement: “Legalization is a huge part of drugs, that’s why there’s shit in them. So yeah, we need everybody involved in the drug world. The government, everything (INTV F5)”.

#### Stigma and trauma

Participants indicated the extremely high levels of stigma that people who use illicit substances experience, impacting the treatment they receive and the way they seek services. Participants reported being treated inhumanely in a number of settings including hospitals, as we heard: “Because the moment they find out you’re an addict they treat you like shit. So, it’s not good. It’s like people still need healthcare too. We’re not like a diseased rabid animal, you know? (INTV F12)”. Furthermore, participants reported high levels of trauma for themselves, their families, friends and communities as a result of the overdose crisis. Participants talked about the trauma of experiencing overdose, witnessing overdose and losing loved ones including children and parents as this quote starts to capture:

I know a lot of people that have died. A lot. You know, like a lot. It’s unbelievable how many people I know that have gone down. It just fucks me up big time. Because I don’t deal with it very well. …Too much of it, and like too close to me. And friends, and family, and partners, and children. You know? I lost a lot of people in the last – just a short period of time there. A lot. It just fucks me up big time. I can’t even watch tearjerker commercials, nothing. Fuck, you know. I’ll just start bawling (INTV F15).

These high levels of stigma and trauma can act as deterrents to accessing services if people fear poor treatment. Drug checking services need to be trauma-informed services and carefully attend to these issues to ensure they are not reinforcing stigma and creating further trauma.

#### An unpredictable and toxic illicit market

Participants overwhelmingly reported a fear about what is in the current drug supply. They reported that there was no way to tell what they were buying as this person indicates: “it’s like Russian roulette nowadays. You never know what you’re buying (INTV F2)” while another stated: “nobody knows what is what, if their heroin is heroin or if their coke is coke, no one knows and it gets really scary (INTV F1)”. There was a frequently voiced belief that fentanyl was in all substances as this person stated: “But it’s not just in the heroin either, it’s in everything (INTV F9)”. At the same time, concerns were not limited to fentanyl; many feared the inclusion of other ingredients that they were not aware of and/or wanted to avoid. Participants mentioned fear of other actives or ingredients such as rat poison, bath salt, battery acid and pig dewormer, with one participant indicating it would be helpful to know “Oh my god, there’s tar in this stuff, or, there’s battery acid in this stuff (INTV F5 )”.

Within the context of an unsafe drug supply, criminalization, stigma and trauma from overdoses and deaths, participants noted little protection or power for people who use or buy substances or accountability for those who sell substances. Moreover, these contextual factors interacted so that some felt targeted with one participant describing the overdose crisis as “a cull (INTV F15)” and another suggested some think of it as a “conspiracy to poison this demographic of people (INTV F5)”. Hence, drug checking can fill an important role by identifying what ingredients are present in a substance that can provide a measure of relative safety within this context.

Thus, participants identified strong potential barriers that highlight how critical it is to understand service user needs and resources and external policies and guidelines that can impact implementation (Table 2). Drug laws and regulations and resulting criminalization and stigma threaten implementation and uptake of drug checking services. As drug checking services are being implemented in a context of stigma, trauma and other risks, establishing trust and safety are essential to the implementation process.

**Table 2:** Findings by CFIR domain and primary constructs

CFIR domain	CFIR construct	Findings
Outer setting		
[Contextual factors that influence successful implementation]	Patient needs and resources	Stigma, trauma and criminalization; unpredictable illicit market; diverse population and needs
	External policy and incentives	Criminalization of drugs and people who use drugs
Intervention characteristics		
[Key attributes of drug checking a harm reduction intervention that influence successful implementation]	Relative advantage	Low barrier and greater benefits than risks of criminalization
	Evidence strength and quality	Accuracy of drug checks providing information on composition using a small sample
	Design quality and packaging	Free and fast service
	Adaptability	Harm reduction messaging
Inner setting		
[Key attributes of drug checking services and sites that influence successful implementation]	Culture and compatibility	Safety; respect; confidential and anonymous service; and harm reduction mandate
	Available resources	Multiple settings to meet diverse needs; paired with compatible services; pharmacies; mobile services
Characteristics of individuals		
[Key attributes of drug checking staff that influence successful implementation]	Knowledge and beliefs	Skilled technicians and trust, respect and freedom from judgement
Process of implementation		
[Key attributes related to the process of implementing drug checking services that influence successful implementation]	Engaging and external change agents	Engaging people with lived and living experience in implementation and the intervention

### Intervention characteristics

Participants shared their perceptions of the key attributes of a drug checking service that would influence successful implementation. Participants expressed that drug checking needs to provide accurate information on composition and be free, fast and require a small sample. Moreover, results need to be communicating according to harm reduction principles. These characteristics are needed in order for the perceived benefits of drug checking to outweigh the substantial risks in the outer setting identified by participants and adapt the intervention to the needs of this diverse population.

#### Accurate information on composition

Given the prevalence of stigma and criminalization of illicit drug use, participants indicated drug checking services would need to be deemed by users as sufficiently worthwhile and informative for people to risk accessing them. They reported it would be critical that drug checking provide information that is accurate and useful, with most participants looking for more information than could be provided by a fentanyl test strip. As this participant stated, they want “to know how much of what is in the drug (INTV F13)” as opposed to whether or not it was positive for fentanyl only*.* People wanted information on the composition and potency of the substance, including other active ingredients or cuts that may be present including but not limited to fentanyl. When asked what would be important for testing, this person expanded: “Accuracy, of course. Accuracy of exactly what was in it, especially the cut, the different kinds of cuts, that’s really what I’d want. If possible, if they could find that out (INTV F1)”. This includes information on purity, strength and amount, as we heard: “I would hope to be able to find out how much of what I’m looking for. For example, how much caffeine is in the dope? How much heroin is actually in the dope? Is it 5%? Is it 2%? (INTV F2)”. These insights highlight that potential service users are interested in knowing the composition of substances tested to prevent overdoses but also to know what is being consumed.

#### Free and fast services on a small sample

Potential costs and time for drug checking were noted as potential barriers. Participants noted that services would need to be free or low cost as this person stated: “Well no charge. I don’t think there should be a charge for it because nobody would be able to afford to pay for it (INTV F2)”. This is not surprising given that the sample was composed mainly of people living on very low incomes or social assistance. Further, many indicated that people would be unlikely to wait long for results noting half an hour as an upper limit:

I think human nature, people want it to be pretty instantaneous, especially if it’s drugs that they’re wanting to use. If they are an addict, they’re going to want to use those drugs right away so they’re not going to wait like an hour or whatever. They’re going to want to know right then and there pretty much, you know, what’s in their drugs and then go and use them. So the shorter the time period, the better (INTV F17).

Other barriers expressed by participants were concerns as to how much of the substance would be needed for testing and whether the sample/substance would be returned after testing. One participant stated: “I would hate to bring my drugs in to a drug checker and they say, ‘Oh yeah, it’s all ketamine so we’re just going to keep it and we’ll dispose of it (INTV F2).” It’s important to recognize that participants purchased drugs with limited resources and thus the substance is a precious resource not to be wasted.

#### Harm reduction messaging

Participants noted that the communication of drug checking results would need to be accessible to meet the different needs of the diverse population that uses substances. Specifically, messaging would need to reflect harm reduction principles of being non-judgemental, pragmatic and neutral. The messaging of results needs to respond to different levels of literacy as well as different amounts of knowledge and experience of substances and substance use.

Furthermore, participants expressed wanting more information than just what drugs are present and in what percentages, they want information about how these drugs and any fillers impact people, or as one person stated: “what it does to you (INTV F7)”*.* They further suggested a service similar to a pharmacy explaining: “like if you pick up a prescription, you know, it comes with the “what it does.” Something like that, with whatever was in your sample (INTV F7)”. We heard how providing drug checking results as harm reduction information would include information that a drug user could actually use to inform choices such as:

Actually go further than just the, “What drug is in this. What else is there in here?” Right? You know what I mean? Like just keep people informed, what’s going on, information is the best highway right? Tell them what’s going on. Then the choice is yours. That’s a so called ‘adult’ (INTV F14).

Drug checking services thus need to have sufficient expertise and technology to provide accurate information on composition on a very small sample, in a fast, effective and efficient manner, to make the service deemed sufficiently useful. Messaging needs to be respectful, pragmatic and informed, offering accurate information on composition as well as specific harm reduction information related to the composition. This is necessary to increase the relative advantage of the intervention as a potential harm reduction response for service users and suitably adapt to meet their needs (Table 2).

### Inner setting

The inner setting is related to the outer context as it often describes how the intervention can mediate the outer contextual factors. Participants described what was needed in a drug checking service from their perspective. Potential service users indicated that multiple settings are needed to meet the needs of this diverse population and that the most critical aspect is to implement drug checking as a safe, respectful harm reduction service within this larger context of criminalization, stigma and trauma.

#### Services for all

Overall, participants indicated the type of setting that would work well would vary depending on the individual and their needs. For those already accessing other harm reduction services, pairing with compatible services would be a facilitator to accessing and sustained use of services. This includes services such as supervised injection sites and overdose prevention sites that have established trust within the community and an adjunct to offering free supplies. For example, one participant noted drug checking should be offered “those places where you go in to use. You should be able to go in and get your dope checked there (INTV F16)” while another recommended an existing service because “A lot of people already use the services, and there’s a trust factor built up, and they feel safe going there (INTV J1)”. Similarly, integrating with services that meet other needs such as providing food, shelter, information on housing, medical care, treatment, counselling services and support networks were noted as facilitators.

Other suggested sites for reaching different populations included pharmacies, supported housing buildings, drop in centres and medical clinics or emergency rooms provided they were confidential and anonymous and did not impact other care such as treatment or prescriptions. One participant discussed this with respect to a medical clinic offering opioid substitution therapy:

Somewhere like this would work well, yes, because obviously the doctors here are trained in addiction and you’re here for a reason. So they are aware of the fact that you’re using drugs. The only thing, barrier, I could see to it is people would be worried that if their doctor was to find out that they were using drugs on the side as well as their prescribed drugs, then that would be an issue – or a barrier – that would be for people to come in and want to test their drugs (INTV F17).

Many participants indicated they would like to be able to test substances themselves, in their own home as this participant stated: “If I had something at home to do myself, I would check it every time. Absolutely (INTV F16)”.

We heard how services would need to be offered in multiple locations to be close to where people live and to address transportation issues including for those who live outside the centre of town and/or rurally. Mobile services were suggested by many as a way to reach those who did not wish to access services for fear of being identified, or with access issues as this quote discusses: “a mobile outreach willing to come and check it really quickly, like when so-and-so is at work and you don’t want them to know (INTV F2)”. Participants also indicated that hours would need to be extensive, flexible and ideally offered “twenty-four hours (INTV P5)” because as another participant stated: “this lifestyle is twenty-four hours. It doesn’t stop, you know what I mean? And it’s random, it’s never, there’s no schedule to it, so, I would honestly try to make it twenty-four hours (INTV B2).”

#### Confidential, anonymous and safe

Regardless of particular needs, participants identified that respectful services that are confidential and anonymous were essential to facilitating access and establishing trust. We heard that many potential service users would not want friends, parents or other family or employers to know about their use and that services would need to account for this. As this participant expressed: “I know that there are some people who are still in the closet and don’t want anyone to know, they’re embarrassed or ashamed or whatever (INTV F2)”.

While participants indicated that integrating services within trusted harm reduction sites would work for some, we also heard how these sites could be a barrier to access for others. Participants noted how many people feared being identified as substance users or going to places associated with use including harm reduction services. One participant stated: “So if there was a way to do it well, so it was accessible to people who don’t necessarily want to go down and have their face seen and everything… Yeah, some way to make it a little more anonymous I guess (INTV F17)”.

Thus, the inner setting would need to address the barriers of criminalization and stigma identified in the outer setting with policies and practices that assures services are both anonymous and confidential. Specifically, having to identify yourself or any type of surveillance would deter use. One participant described: “you wouldn’t have to be telling who you were, and it wouldn’t have cameras, it’d be a safe, secure, private location (INTV F7)”. Another participant defined what a confidential service would look like saying: “confidentiality is being able to go somewhere to ask your questions without the risk of it, um…causing other issues in your life…being completely confidential and about just the health aspect of it (INTV F17)”.

Participants noted how fear of repercussions could pose a barrier. While there were variations, overall, participants expressed how implementation would fail if located in settings perceived as punitive to people who use drugs. This included hospitals as this participant states: “I don’t think at the hospital, I don’t think I would take my drugs to the hospital because they could call the cops (INTV F4)” as well as places with police involvement: “First on the top of my mind would be police station, don’t have it there (INTV B1)”. One participant provided this guiding principle: “I think if the authorities were involved, then nobody would really want to go there (INTV F7)”*.* Thus, drug checking services need to take care to assure relative safety within the context of criminalization.

#### Harm reduction mandate

A common theme that emerged was that the organizational culture of drug checking needed to promote personal harm reduction practices rather than seek to operate as a deterrent to drug use. For many, stopping drug use was not an aim or reason to access drug checking; rather, the benefit would be accessing information about a substance to inform use. One participant expressed fear about the unpredictable toxic drug supply and the limited options to use more safely, saying: “I was so scared of using drugs, I just didn’t know what to do, and I didn’t want to quit. I was cutting down, but quitting right now is not an option for me. It’s just not something I’m interested in (INTV F1)”. Participants noted people may choose to use a substance regardless of what is in it, particularly if they are in withdrawal: “I know when I’m so sick, I have to have something. I have to get something, so I just do it anyway (INTV F2)”. Furthermore, while some are trying to avoid fentanyl others may seek it out as this person states about fentanyl: “I’ve found a lot of people, that’s actually what they look for (INTV F13)”.

This illustrates the importance of a harm reduction approach that does not make assumptions about how information might be received or seek to change behaviour, and non-judgemental messaging. Moreover, it is critical to recognize that what might be perceived as choice in such situations, such as which substance a person uses, is driven by broader factors such as marketing and availability. Overall, we heard that when implementing drug checking in a context of stigma, trauma and criminalization, how services are implemented matters greatly. The organizational culture must be compatible with values, practices and policies that are respectful, confidential and anonymous and ensure safety, and support service users to practice their own harm reduction practices (Table 2). Further, multiple settings are needed to meet service users’ needs which requires careful consideration and resourcing (Table 2).

### Characteristics of individuals

Participants were asked who should be providing drug checking services as well as who should be part of the implementation processes. They noted that how staff treated service users was of primary importance, emphasizing that trust, respect and freedom from judgement are essential to counter the stigma associated with substance use. They also recognized that skilled technicians would be required.

#### Trust, respect and freedom from judgement

“The judgement, right away as soon as you walk through the door, is the biggest barrier (INTV F4)” was a sentiment expressed in many ways from many participants. Participants feared being treated poorly and indicated “being belittled or judged for being an addict (INTV F12)” would be a critical barrier to accessing or sustained use of drug checking services. Overall, it was reported that one of the most essential aspects for the service would be staff who treated them with respect and dignity, did not judge them and could be trusted to maintain confidentiality:

I’d like the place that I can come to and not, not get judged for being a drug addict or being a person that does drugs, and they don’t even talk to you like you’re a person, and talk to you like how people should be talked to… Like they say it’s confidential, a lot of, some places they’re not. They say it’s confidential and then they go behind your back and talk to the people (INTV F11).

They further expressed that a barrier to using the services would be “if they didn’t treat us like human beings, they treated us like animals, or like just a number (INTV F11)”.

Peer workers and people with lived experience were frequently suggested as a good fit as they would feel comfortable and safe with them. However, participants also noted others such as doctors, nurses, pharmacists or social workers could all be appropriate provided they treated them with respect and could be trusted to maintain their privacy. A common theme was that it was more important how staff treated people, rather than their background. As one person stated: “its not who they are but how they, how they treat us, and whether it’s a safe place to go (INTV B2)”. The quote below reflects on who should be involved in providing such services and the primacy of being treated with respect and without judgement rather than the actual provider:

What feels comfortable to me? So I was going to say the number one thing that comes to the top of my head is people who have had experience with drugs themselves, but it doesn’t necessarily have to be that way. I guess that would help, but again, people who respect anonymity. They don’t go around saying, “Oh, guess who I saw today!”… And not the judgement, non-judgemental. People with some compassion and heart. People look at people like us, people who are using drugs or even people who are clean from drugs, look at us like a problem. … People who look at us like we’re just human beings. People who see us for that (INTV F3).

#### Skilled technicians

Participants also noted that skilled technicians would be necessary. Some participants noted that drug checking would require individuals with a unique skill set if using spectrometers. One participant assumed the need for such a technician stating: “the person that knows how to work the tester, of course…a pharmacist, or someone into chemistry, or something like that (INTV F7)”. Similarly, another participant expressed staff “should be a chemist” defined as a person who “can break it right down and say, right down to the percentage…how good it is, he can tell you (INTV F9)”.

Skilled staff with adequate training are also required to be able to provide the type of harm reduction information that service users want about substances. Thus, in addition to instrument technicians, a solid background in harm reduction and substance use is necessary to provide information that is most useful. Thus, an optimal drug checking team might include both a skilled technician and a peer or harm reduction worker.

This highlights the importance of the knowledge and beliefs of those implementing and operating the intervention, specifically both the necessary skill in operating the intervention as well as the attitudes and approaches of the individuals to the service user and the service being consistent with principles of harm reduction (Table 2). Respectful treatment by all staff, technicians or otherwise, is critical in helping to create safe and welcoming environments. This can help to establish trust for service users necessary with issues around stigma and trauma and criminalization.

### Process of implementation

Participants were asked how drug checking could and should be implemented. As noted above, participants identified trust as critical to implementation success and that engaging people with lived and living experience in implementation was a strategy to facilitate that trust and create meaningful inclusion for people who use substances.

#### Engaging people with lived experience

Engaging peers or people with lived experience emerged as a powerful strategy to address many of the identified barriers related to criminalization and stigma and perceptions of safety. Participants noted that word-of-mouth feedback from people who use the service, how they were treated, and whether it is working for them would be important. As this person stated: “word of mouth from someone I knew and trusted (INTV F13)” would be required before accessing the services. Until then, participants expressed the implementation process could be slow, saying: “Yeah, well at first it’s going to be very, um, standoffish I think. It will take a while (INTV F14)”.

Many suggested peers and those with lived experience as suitable staff that would make them feel comfortable using the service and facilitate trust as this participant highlighted when discussing peers working at a supervised consumption site:

I always think places like this should involve like, drug users.…I think it should be managed and supervised and whatever from people of this sort that are running this place, but also people that are active in the street and like actually, people that are—but you know, they have to be respectful with reason. You’d have to, you’d have to choose them right. But I think, like you know, people like I or myself and like other people should also be involved with it too. It helps. It brings trust. You know what I mean? To see like, you know, someone that you know and like talk to on the streets daily, like, to be there too, right? People trust that. People, it’ll, it’ll help get people there and stuff. Right? And feel safe there, so (INTV B2).

Furthermore, some noted that they would welcome the opportunity to be involved, and that meaningful work would help improve their own lives.

This highlights the need for meaningful inclusion and representation of peers and people with lived experience in different aspects of the implementation process in order to develop services that meet people’s needs and establish trust for drug checking services. Thus, engaging peers as external change agents is a critical strategy for building trust and inclusion of peers as staff can contribute to healthier communities (Table 2). These are critical components of developing services that align with principles of harm reduction and health equity (Table 2).

## Discussion

This study asked potential service users how best to implement community drug checking services as a response to an unpredictable and frequently toxic unregulated drug supply, and the high rates of overdose in communities. We heard a readiness for the implementation of drug checking as a harm reduction response that is able to provide accurate information on composition of substances, as well as harm reduction information to bolster one’s own harm reduction strategies. Within this context, we heard that criminalization, stigma and trauma pose the most significant barriers to service use. Staff and services must strive to create trust and safety through provision of respectful and confidential services that operate according to harm reduction principles. Engaging people with lived experience is one strategy that can help create trust. Varied locations and neutral, non-judgemental messaging are required to meet the needs of the diverse population that may benefit from drug checking.

External contextual barriers present substantial challenges to implementation and to the potential effectiveness of community-wide drug checking services. Overwhelmingly, we heard how criminalization of drugs and people who use and/or sell drugs is the most significant barrier to the implementation process and an intervention’s success. As noted by studies exploring health care for people who use drugs [40–44], the extent to which the implementation of drug checking can respond to and mitigate the risks of being criminalized and stigmatized appears most critical to the acceptability and success of community drug checking. Confidential and anonymous services, and how well the culture and compatibility of the service, setting and staff align with harm reduction principles and practices, is critical.

Within this context, in order for the relative advantage of drug checking to be sufficiently high, the perceived benefits of accessing drug checking services need to exceed the significant risks of criminalization and stigmatization. Similar to research in Vancouver, BC [45], for the relative advantage of drug checking to be high enough to outweigh these risks, participants wanted accurate, precise and detailed information on composition of substances and harm reduction. As noted in other research [18, 46], participants expressed that the value of drug checking would be in providing new information so they could have the most knowledge to further inform their drug use and personal harm reduction practices. Similar to the FORCAST research in the USA [19], we heard this requires designing an intervention that includes skilled technicians to operate sophisticated technology that provides results with a high degree of accuracy and detail, that are pragmatic and useful (such as potency), for those accessing the services. Furthermore, drug checking results require harm reduction messaging that does not presume or judge how a person acts with that information [18].

Our study highlights that relevant outcomes for drug checking within this context need to be carefully considered. We heard that drug checking is useful for gaining knowledge relevant to using drugs and informing one’s own harm reduction and that non-use or disposal is not a goal or option for many. Within this context, the desired outcome of drug checking therefore cannot be the disposal of one’s drugs, or not taking the drug being tested, as has been utilized as an outcome in some previous research within dance and festival drug checking services [15, 47–49]. Such outcome measures for community drug checking can minimize the value of drug composition evidence to further inform drug use and harm reduction. Our study concurs with findings from recent Canadian and American research teams that discarding one’s drugs is a much less relevant and respectful service objective for community drug checking services seeking to reach people impacted by poverty, homelessness and other structural vulnerabilities where it may not be feasible for many to discard and purchase from another source [8, 18, 45, 46]. Further research is needed to better understand the benefits and impacts of drug checking within these contexts that take this into account.

Participants also indicated the need for quick services, multiple sites and extensive hours to adequately serve the complex needs of a diverse population. This included suggestions for mobile drug checking services that travel to communities. The extent to which community drug checking can be adaptable to reach anyone who uses unregulated drugs is a significant factor influencing its potential impact as a harm reduction response. Participants expressed that while not everyone uses drugs, anyone does. Implementing services to reach the socially diverse and geographically dispersed populations of people that use unregulated drugs is recognized as a significant implementation challenge in Canada [50, 51]. The sites in which drug checking is located and accompanying services will impact which people may access the service and which people will not. How the services are designed and promoted and who provides the services are further factors to consider. Given the current technologies and drug checking methods available, and the level of experience and time required to operate these, these could very well present conflicting needs. Research is needed to investigate low cost, portable technologies that can be operated without extensive training.

People with lived and living experience of drug use are integral to the success of the implementation process and to interventions’ ability to provide trust and respect to people seeking drug checking services. This finding resonates with other studies in which trust has been identified as a key factor influencing access [41, 52–54]. We also heard how the most significant factor was that individuals involved be respectful regardless of if they identify as a person who uses drugs or not; this aligns with recent findings of a fentanyl test strip pilot project [55]. Moreover, given the high levels of trauma experienced as part of the overdose crisis, a trauma-informed approach [56] is required for service delivery. Therefore, while there is an assumption that drug checking benefits from the unique skills of chemists or pharmacists and others with technical skills in operating spectrometers and interpreting chemometric data, collaboration with people with lived experience with drugs and harm reduction backgrounds is equally valuable.

Although the findings from this research can inform community drug checking implementation, the results also confirm the significant harmful impacts of criminalization of drugs and people who use/sell drugs and how criminalization negatively impacts the implementation of wanted harm reduction responses [57–60]. As also expressed by Bardwell et al. [45], implementation of drug checking is compromised by the criminalization of people who are to access the services and the sample they seek to check. As stated by Palamar et al. [61], drug checking continues to exist within a legal grey zone that creates mistrust from potential service users and barriers to the implementers of these public health, harm reduction services [18, 61, 62]. Thus, there is value in operating drug checking as a government sanctioned response and changes to the drug laws are critical for a safer, more regulated drug supply.

A strength of the study is that to understand the current context and how best to implement services that are acceptable and meet service user needs, it sought the perspectives of people who use substances. Furthermore, our collaborative research team included community partners. The study was based in Victoria, BC, Canada, where the burden of the overdose crisis is extremely high. Further, we used a well-established implementation framework to explore this unique context and potential barriers and facilitators for implementation across a number of domains known to be relevant to implementation success. This study also has several limitations. Often, research participants had little knowledge of and no experience with drug checking which may have limited their input as to how to implement this novel intervention. Recruitment within harm reduction sites would have limited our reach to at-risk populations not accessing these services. Future research on community drug checking that focuses on the unique perspectives and needs of the diverse populations impacted by the risks of unregulated drugs is needed including youth and young adults, people with higher incomes, suburban and rural populations, Indigenous peoples, women and those identifying as queer, transgendered and non-binary, among others. This research reflects a limited place and time and may be less relevant to other contexts.

## Conclusions

The current illicit drug overdose crisis within North America and in other countries requires expanded and new responses as the unregulated drug market includes unpredictable and potentially lethal substances including fentanyl analogues. Community-wide drug checking is a potential intervention being increasingly explored as one such public health response. Our findings confirm how drug checking is implemented matters greatly to its success and that there are significant barriers and critical factors that influence implementation and an intervention’s success. We found that implementing community drug checking faces significant challenges within the current context of criminalization and stigmatization of drugs and people who use/sell drugs, and the ongoing traumas experienced by potential service users. The benefits of accessing drug checking services need to exceed the significant risks of criminalization and stigmatization. Drug checking services should seek to provide accurate and detailed information on composition of substances, as well as harm reduction information to inform (rather than deter) use. The culture and compatibility of the service, setting and staff to harm reduction principles and practices is critical.

## Data Availability

Data are not available due to the sensitive, confidential and potentially incriminating nature of the data.

## Abbreviations

CFIR: Consolidated Framework for Implementation Research
BC: British Columbia
SPOR: Strategy for Patient-Oriented Research
POR: Patient-Oriented Research
CBPR: Community-Based Participatory Research (CBPR)
